# Relationship between Swimming Ability of College Students and Their Regular Exercise Habits

**DOI:** 10.3390/sports10100145

**Published:** 2022-09-26

**Authors:** Feng-Hua Tsai, Hsiu-Tao Hsu

**Affiliations:** Center for Physical and Health Education, Si-Wan College, National Sun Yat-Sen University, Kaohsiung 804201, Taiwan

**Keywords:** college students, swimming ability test, exercise habit, sports policy

## Abstract

Background: This study examined the relationship between the five-level swimming ability of college students and their regular exercise habits. Methods: This study applied to Academia Sinica for permission to use the raw data from the Survey on the Sports Participation Situations of Students in All Levels of Education, which was conducted by the Sports Administration, Ministry of Education, Taiwan; a secondary data analysis of the obtained data was then conducted. Results: Overall, 18,515 pieces of data were selected for analysis, and the results revealed that 85.9% of the surveyed students had learned to swim; those who had regular exercise habits exhibited a higher level of swimming ability than those without such habits. A logistic regression analysis showed that progressing to a higher level of swimming ability increased the likelihood of having regular exercise habits by 20%. Conclusions: The present study showed that level of swimming ability was significantly correlated with regular exercise habits. In the future, exercise self-efficacy theories can be applied to explore the relationship between exercise skill proficiency and regular exercise habits.

## 1. Introduction

Taiwan is situated in the subtropical zone and surrounded by oceans, has an excellent environment for many aquatic recreation activities. For the past 20 years, Taiwan has promoted various water safety and swimming ability-related policies and developed marine culture and water-friendly education, thereby improving the personal safety, drowning prevention, and self-rescue skills of the Taiwanese people and reinforcing their conceptual knowledge of water safety [1]. The promotion of swimming-related water activities not only features health-improvement implications but also manifests the geographical advantages and characteristics in Taiwan. For sports promotion and marine education, swimming has become one of the top 10 leisure sports in Taiwan [2]. In 2006, swimming ability tests were implemented for elementary and junior high school students. Swimming ability was initially divided into 10 levels and, since 2010, into five levels. The five-level swimming ability indicators incorporate self-rescue skills and swimming skills and are established in accordance with the water competence framework (i.e., three water-friendly skills, namely awareness of water safety, basic swimming skills, and ability to help others) promoted by Stallman (2017) [3]. At the time of writing, schools in Taiwan have adopted the five-level swimming ability indicators stipulated by the Ministry of Education as criteria for assessing the swimming ability of students.

Adequate physical activity allows individuals to maintain their physical fitness and enhance their mental health [4,5,6]. For sport policies, Taiwan’s government has been focused on methods for increasing the proportion of Taiwanese people with regular exercise habits. The results of official surveys indicated that when students progress academically (e.g., from elementary school to university), the proportion of students with regular exercise habits decreases [7,8]. Studies have also shown that having regular exercise habits during school semesters is highly associated with having lifelong regular exercise habits [9,10,11,12]. Haerens et al., (2010) indicated that in addition to taking courses or participating in extra-curricular sports during their time as students, personal preferences, interests, and experience in specific sports influence individuals’ ability to develop regular exercise habits [13]. Furthermore, numerous sports-related studies have explored exercise self-efficacy, which refers to individuals’ confidence in achieving a specific exercise goal or overcoming obstacles to developing regular exercise habits. A study indicated that exercise self-efficacy is positively correlated with the level of sports participation [12]. Another study on endurance athletes revealed that having memorable sports experiences, overcoming challenges and adversity, and being familiar with one’s own body are key factors that enable such athletes to continue endurance sports [14]. Because of the risks inherent in water activities, learning to swim is an inherent challenge. Therefore, the level of students’ swimming skills tends to affect their recognition of exercise self-efficacy, which in turn affecting their sports participation.

The present study investigated factors that influenced regular exercise habits. In particular, it explored whether the swimming-related policies promoted in Taiwan have led to the establishment of a correlation between the swimming ability levels of students and their regular exercise habits. Because of the feasibility and legitimacy of implementing swimming ability certification, the certification system with five-level swimming ability indicators implemented by the Sports Administration, Ministry of Education, can be applied to enhance swimming ability. On the basis of the influence of a single form of sport competence, the present study collected empirical data related to regular exercise habits to clarify the relationship between swimming ability and regular exercise habits.

## 2. Materials and Methods

### 2.1. Participants

To examine the relationship between the swimming ability of students and their regular exercise habits, the present study conducted a correlation research and performed a secondary data analysis on the basis of a systematic database developed by a governmental agency. The methodology implemented in the present study has reliability and validity and does not necessitate questionnaire reliability and validity analyses [7,8]. For the present study, an application was submitted to the Survey Research Data Archive (SRDA) of Academia Sinica for permission to use the data from the 2018 and 2019 Survey on the Sports Participation Situations of Students in All Levels of Education conducted by the Sports Administration, Ministry of Education, and a secondary data analysis of the data was performed. In the most recent phase of the survey, data on whether the swim ability, swimming ability level, cumulative exercise time per week, and exercise intensity of students were collected. Prior to the administration of the questionnaire survey, The questionnaires used in this study are official government surveys, and data are collected in accordance with standard procedures of “pre-testing” to ensure that the questionnaires have a high degree of reliability and validity [7,8]. The participants were sampled from among day-time college students for the 2018–2019 academic year (students from sports recommendation were excluded). Questionnaires were distributed to the participants by mail, and the participants were sampled proportionally from universities and colleges in Taiwan on the basis of the numbers and gender ration of students in these universities and colleges.

### 2.2. Swimming Ability Level

At various educational levels, schools typically adopt the five-level swimming ability indicators established by the Ministry of Education as the criteria for assessing the swimming ability of students. In the questionnaire survey for the present study, an additional level (i.e., Level 0 refers to individuals who cannot swim) was added to the original five swimming ability levels. Table 1 lists the skills required for each competence level.

### 2.3. Definition of Regular Exercise

In the present study, under the standards stipulated by the Sports Administration for surveys on sports status [2], a regular exercise habit is defined as participation in 30 min exercise three times (days) or more per week at an moderate exercise intensity that causes panting and sweating [2]. Because the participants were students, the general physical education were treated as the exercise time. However, whether the exercise intensity during the physical education classes reached the standards of panting and sweating was determined by the students themselves.

### 2.4. Data Analysis

After the raw data of the 2018 and 2019 survey on the sports participation situations of students in all levels of education (conducted by the Sports Administration, Ministry of Education) were downloaded from Academia Sinica, 19,442 questionnaires were distributed for the present study, and 18,515 responses retrieved (i.e., a return rate of 95.3%).

The present study targeted college students who were college students during the 2018–2019 academic year. For the purpose of the present study, after the question items (e.g., gender, grade level, height, weight, average number of days of exercise per week, exercise intensity, swim learning experience before attending college, ability to swim, and level of swimming ability) with missing responses were excluded from analysis, and 17,105 pieces of data were obtained. Therefore, 92.3% of the raw data were used as valid data in the present study. The collected data were analyzed using SPSS (version 22.0, IBM, Armonk, NY, USA). The statistical methods that were applied were descriptive statistics for the demographics of participants, chi-square testing and one-way ANOVA were used to verify the difference between swimming ability level and the cumulative exercise time per week, and the logistic regression were used to investigate the influences of students’ demographics and swimming ability level on their development of regular exercise habits. The present study investigated the relationship between swimming ability and regular exercise habits. Therefore, only the data collected from students who had learned to swim were included in the analysis by using the aforementioned statistical methods.

## 3. Results

Through an examination of the students’ distribution based on their gender and grade level, the questionnaire survey conducted in the present study was verified to have no sampling bias with respect to a specific gender or grade level. Among the 17,105 college students, 14,685 students (85.9%) self-reported that they had learned to swim; only 2420 students (14.1%) self-reported that they had not yet learned to swim. Table 2 reveals that 28.3% of college students in Taiwan had regular exercise habits. For cumulative exercise time per week, 73.0%, 14.9%, and 12.1% of the students self-reported that their cumulative exercise time was <150 min, 150–299 min, and >300 min per week, respectively. The present study mainly explored the relationship between the swimming ability of college students and their regular exercise habits. Therefore, the subsequent data analysis focused on the 14,685 students who had learned to swim.

**Figure 1:**
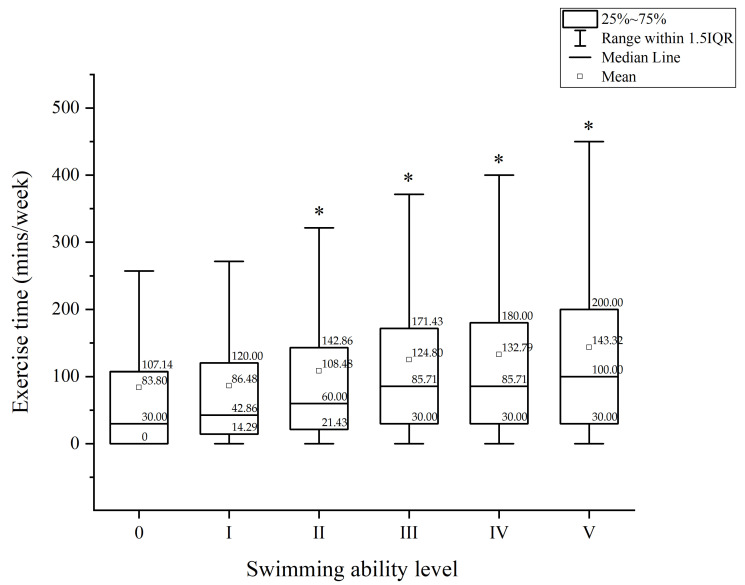
The cumulative exercise time per week for college students with different swimming ability level. The * means that the students with swimming ability level II, III, IV, V had significant longer exercise time than those with swimming ability level 0.

Table 3 indicates that, among the 14,685 students who self-reported that they had learned to swim, the swimming ability of 689, 1774, 1927, 3359, 2783, and 4150 students were at Level 0, I, II, III, IV, and V, respectively. In the present study, cumulative exercise time per week was categorized into three levels, namely <150 min, 150–299 min, and >300 min. The research results displayed that those who exhibited a higher swimming ability level reported a longer exercise time per week, showing that college students with a higher level of swimming ability tended to exercise. In Figure 1, exercise time was expressed in minutes. This figure reveals the differences in cumulative exercise time per week among students with various levels of swimming ability. The cumulative exercise time of students whose swimming ability was at Level 0, I, II, III, IV, and V was 83.77 ± 121.55 min, 86.47 ± 115.22 min, 108.53 ± 126.67 min, 124.75 ± 137.95 min, 132.77 ± 138.33 min, and 143.29 ± 143.74 min, respectively. A one-way ANOVA analysis indicated that individuals’ level of swimming ability significantly affected their cumulative exercise time per week (F = 52.34, *p* < 0.01, power = 1.00). That is, cumulative exercise time per week increased with the level of swimming ability

Finally, to explore whether the college students’ level of swimming ability affected their development of regular exercise habits, a logistic regression analysis was conducted to examine this relationship (Table 4). In addition, the present study also analyzed the influences of gender and age. The results showed that gender and level of swimming ability were significantly correlated with regular exercise habits (odds ratio [OR] = 0.53 for gender; OR = 1.20 > 1 for level of swimming ability); that is, fewer female students had regular exercise habits relative to male students, and an increase in students’ level of swimming ability by one level increased their likelihood of having regular exercise habits by 1.20 times relative to students without a regular exercise habit. However, the grade level (i.e., age) of the students did not significantly affect their development of regular exercise habits.

## 4. Discussion

### 4.1. Survey on Level of Swimming Ability of College Students in Taiwan

The statistical analysis results from the 17,105 questionnaires collected between 2018 and 2019 can be summarized as follows: (1) 85.9% of the surveyed college students self-reported that they had learned to swim at lower grade levels; only 14.1% of the students self-reported that they had not yet learned to swim. Therefore, more than 80% of college students in Taiwan had experience with learning how to swim before they entered college. These results are consistent with Taiwan’s current implementation directions from the Sports Administration, Ministry of Education, with respect to the granting of subsidies for promoting swimming and water sports in schools. Most students had learned to swim in senior high school or earlier. (2) For swimming ability, approximately 70.1% of the surveyed students could swim at least 25 m and had basic self-rescue skills (i.e., Level III or higher level of swimming ability). An international research investigated a self-estimated questionnaire survey of first year collegiate physical education students in Japan, Australia, New Zealand and Norway confirmed that 83.5% students estimated that they could swim nonstop for a distance of more than 300 m [15]. In contrast, a self-report study that examined the swimming ability of US, 43.6% of the adolescents (aged 12–17 years) responded as a “at risk” swimmers (defined as “unable to swim” or “could swim a little, but were not comfortable in deep water”) [16]. In the present study, more than 70% college students had a sound basic aquatic skill, indicating that academic schools in Taiwan have achieved excellent teaching results for swimming and water sports, and that Taiwan’s government policies have helped its people to develop basic water sports and self-rescue skills.

### 4.2. Relationship between Swimming Ability of College Students and Their Regular Exercise Habits

Table 2 indicates that only 28.3% of college students in Taiwan met the standards for regular exercise habits. Therefore, most college students in Taiwan did not have regular exercise habits. Studies have reported that the physical activity of college students is positively correlated with their score on quality of life questionnaires [12,17,18,19]. The present study aimed to identify manipulable influencing factors from the survey results on the sports participation of college students and to use these factors as intervention indicators for improving the sports participation of students. The statistical analysis results from the present study showed that when the surveyed students increased their level of swimming ability, their cumulative exercise time significantly increased. In regard to the relationship between regular physical activity and swimming ability, Lohmus et al. found that an active lifestyle were positively associated with children’s ability to swim. Actively participating in physical activity classes or outdoor activity may reflect to the increased self-efficacy in children about their motor capacity, and it is likely to increase an individual’s success in learning new skills, such as swimming skills [20]. Another research also reported that self-efficacy, self-satisfaction, past performance and personal goal setting had a significant positive relationship between the swimming ability of college students [21]. These findings indicate that the swimming ability could be an objective factor of self-efficacy, also an outcome variable accumulated from past learning. What was interesting about this study was that we found that the better the swimming ability (measurable level), the higher the probability of engaging in regular exercise. A logistic regression analysis revealed that level of swimming ability influenced the development of regular exercise habits. In addition, the proportion of female college students who exercised regularly was only half of that of male students who exercised regularly. This finding is consistent with that of another study [22]. In addition, college students with a higher level of swimming ability were more likely to exercise regularly. Our results revealed that an increase in the swimming ability of a student by one level increased their likelihood of developing regular exercise habits by 1.20 times relative to students without regular exercise habits; this finding suggested that the regular exercise habits of college students were highly correlated with their level of swimming ability. Studies on sports skills and self-efficacy have reported that individuals with higher levels of sports skills have higher self-efficacy [23], and that exercise self-efficacy is positively correlated with sports participation [12]. Although the present study did not investigate students’ self-efficacy, we used the level of swimming ability as a measure of students’ exercise self-efficacy, which was then linked to their regular exercise performance. These findings could explain why the students in the present study who had a higher level of swimming ability were more likely to exercise regularly. With a higher level of education, students were less likely to exercise regularly; however, by acquiring a higher level of sports skills and improving their exercise self-efficacy, students were more likely to leverage their confidence in sports to develop regular exercise habits.

### 4.3. Combination of Sports Skills with Exercise Self-Efficacy Theories to Explore Likelihood of Exercising Regularly

Studies on the sports participation of college students have reported that various factors (e.g., health, sense of accomplishment, time, and social activity) influence whether college students can maintain their exercise habits. Among the reasons that hinder sports participation, time and frustration are the main ones affecting both male and female students (approximately 40–50%). Therefore, a sense of accomplishment and frustration due to exercise substantially influences whether individuals will continue to exercise. Snedden et al., (2018) indicated that college students with higher levels of sports skills and physical activity are more positive and healthier psychologically [17]. In the present study, swimming (a common sport among Taiwanese students) was explored on the basis of exercise self-efficacy theories, and the results revealed that the level of swimming ability of college students was highly correlated with their regular exercise habits. The results of the present study indicated that level of sports skill can be incorporated into a theoretical framework for exercise self-efficacy to explore how this factor can influence regular exercise habits. However, because the present study adopted a cross-sectional exploratory design, it could not verify or clarify the factors (e.g., sports skill such as level of swimming ability or other factors such as learning environment, family background, and physical education course/knowledge) that substantially influenced the development of regular exercise habits, and this is a topic that warrants further studies.

## 5. Conclusions

The present study showed that level of swimming ability was significantly correlated with regular exercise habits. Although more research is required to verify whether improving sports skills can facilitate the development of regular exercise habits among students, the empirical data collected in the present study can be combined with various theories to establish a foundation for further studies.

## Figures and Tables

**Table 1:** Swimming Ability Level.

Level	Swimming Ability	Self-Rescue Skills
0	Cannot swim at all	
I	Pick up objects in water twice. Kick the wall to float in the water for 3 m, and stand up.	Stand up and rhythmically breathe 20 times. Perform jellyfish floating for 10 s (without breathing).
II	Use legs to paddle and move forward 10 m. Swim 15 m (breathe more than three times).	Use a buoyancy aid to float for 60 s. Perform jellyfish floating for 20 s (breathing allowed). Perform back floating for 15 s.
III	Swim 25 m (breathe more than five times).	Perform jellyfish floating for 30 s (breathe every 10 s). Perform back floating for 30 s.
IV	Swim backstroke, breaststroke, butterfly stroke, or front crawl stroke for 50 m (the swimmer must turn around without touching the ground).	Tread water for 30 s. Perform back floating for 60 s.
V	Continuously swim 100 m (the swimmer must turn around without touching the ground).	Tread water for 60 s. Perform back floating for 120 s.

**Table 2:** Demographics of Participants.

	Number	Percentage %
Total	17,105	-
Gender		
Male	8253	48.2
Female	8852	51.8
Grade		
1	4704	27.6
2	4639	27.2
3	3901	22.9
4	3798	22.3
Learned to swim		
Yes	14,685	85.9
No	2420	14.1
Regular exercise		
Yes	4928	28.3
No	12,475	71.7
Cumulative exercise time per week		
<150 min	12,710	73.0
150–299 min	2589	14.9
>300 min	2104	12.1

**Table 3:** Correlation between Swimming Ability Level of College Students and Their Cumulative Exercise Time per Week.

		Cumulative Exercise Time per Week			χ^2^	*p*
		<150 min n = 10,775	150–299 min n = 2285	>300 min n = 1857		
Swimming ability level	N					
0	689	571 (82.9)	63 (9.1)	55 (8.0)	252.727	<0.01
I	1774	1465 (82.6)	182 (10.3)	127 (7.2)		
II	1927	1470 (76.3)	270 (14.0)	187 (9.7)		
III	3359	2431 (72.4)	512 (15.2)	416 (12.4)		
IV	2783	1927 (69.2)	452 (16.2)	404 (14.5)		
V	4150	2731 (65.8)	777 (18.7)	642 (15.5)		

**Table 4:** Logistic Regression Analysis of Influences of Students’ Demographics and Level of Swimming Ability on Their Development of Regular Exercise Habits.

Factor	B	SE	Wald’s χ^2^	OR (95% CI)	*p*
Gender	−0.63	0.04	275.83	0.53 (0.50–0.57)	<0.01
Grade	−0.02	0.02	1.254	0.98 (0.95–1.01)	0.263
Swimming ability level	0.18	0.01	194.61	1.20 (1.17–1.23)	<0.01

## Data Availability

The data presented in this study are available on request from the corresponding author.

## References

[1] Lwun-Syin L., Cheng-Chieh C. (2019). The practice of marine education issue-based curriculum integration in the 12-year basic education. Sch. Adm..

[2] Sports Administration, MOE (2020). 2020 Sports Status Survey Report.

[3] Stallman R.K. (2017). From swimming skill to water wompetence: A paradigm shift. Int. J. Aquat. Res. Educ..

[4] Biddle S.J., Asare M. (2011). Physical activity and mental health in children and adolescents: A review of reviews. Br. J. Sports Med..

[5] Kim E.S., Kubzansky L.D., Soo J., Boehm J.K. (2017). Maintaining healthy behavior: A prospective study of psychological well-being and physical activity. Ann. Behav. Med..

[6] McMahon E.M., Corcoran P., O’Regan G., Keeley H., Cannon M., Carli V., Wasserman C., Hadlaczky G., Sarchiapone M., Apter A. (2017). Physical activity in european adolescents and associations with anxiety, depression and well-being. Eur. Child Adolesc. Psychiatry.

[7] Sports Administration, MOE (2020). 2018 Students Participate in All Levels of Education (ai020011).

[8] Sports Administration, MOE (2021). 2019 Students Participate in All Levels of Education (ai020012).

[9] Bailey R. (2005). Evaluating the relationship between physical education, sport and social inclusion. Educ. Rev..

[10] Chen S., Snyder S., Magner M. (2010). The effects of sport participation on student-athletes’ and non-athlete students’ social life and identity. J. Issues Intercoll. Athl..

[11] Hsieh S., Tsai J., Chang S., Cheng C., Sung Y.T., Hung T. (2018). The relations between 3-year changes in physical fitness and academic performance in nationally representative sample of junior high school students. Sci. Rep..

[12] Joseph R.P., Royse K.E., Benitez T.J., Pekmezi D.W. (2014). Physical activity and quality of life among university students: Exploring self-efficacy, self-esteem, and affect as potential mediators. Qual. Life Res..

[13] Haerens L., Kirk D., Cardon G., De Bourdeaudhuij I., Vansteenkiste M. (2010). Motivational profiles for secondary school physical education and its relationship to the adoption of a physically active lifestyle among university students. Eur. Phys. Educ. Rev..

[14] Anstiss P.A., Meijen C., Marcora S.M. (2020). The sources of self-efficacy in experienced and competitive endurance athletes. Int. J. Sport Exerc. Psychol..

[15] Moran K., Stallman R.K., Kjendlie P., Dahl D., Blitvich J.D., Petrass L.A., McElroy G.K., Goya T., Teramoto K., Matsui A. (2012). Can you swim? An exploration of measuring real and perceived water competency. Int. J. Aquat. Res. Educ..

[16] Irwin C.C., Irwin R.L., Ryan T.D., Drayer J. (2009). Urban minority youth swimming (in)ability in the united states and associated demographic characteristics: Toward a drowning prevention plan. Inj. Prev..

[17] Kruger S., Sonono E. (2016). Physical activity and psychosomatic-related health problems as correlates of quality of life among university students. J. Psychol. Afr..

[18] Krzepota J., Biernat E., Florkiewicz B. (2015). The relationship between levels of physical activity and quality of life among students of the university of the third age. Cent. Eur. J. Public Health.

[19] Snedden T.R., Scerpella J., Kliethermes S.A., Norman R.S., Blyholder L., Sanfilippo J., McGuine T.A., Heiderscheit B. (2019). Sport and physical activity level impacts health-related quality of life among collegiate students. Am. J. Health Promot..

[20] Lohmus M., Osooli M., Pilgaard F.I.H., Ostergren P.O., Olin A., Kling S., Albin M., Bjork J. (2022). What makes children learn how to swim?—Health, lifestyle and environmental factors associated with swimming ability among children in the city of Malmo, Sweden. BMC Pediatr..

[21] Τheodorakis Y. (1995). Effects of self-efficacy, satisfaction, and personal goals on swimming performance. Sport Psychol..

[22] Pauline J. (2013). Physical activity behaviors, motivation, and self-efficacy among college students. Coll. Stud. J..

[23] Tan Q., Shao W. (2021). Investigation on health promotion by the typical sports for teenagers with self-efficacy and sports commitment questionnaires. Evid.-Based Complement. Altern. Med. ECAM.

